# A QR Code for the Brain: A dynamical systems framework for computing neurophysiological biomarkers

**DOI:** 10.21203/rs.3.rs-4927086/v1

**Published:** 2024-09-18

**Authors:** William Bosl, Michelle Bosquet Enlow, Charles Nelson

**Affiliations:** University of San Francisco; Boston Children’s Hospital; Harvard Medical School

## Abstract

Neural circuits are often considered the bridge connecting genetic causes and behavior. Whereas prenatal neural circuits are believed to be derived from a combination of genetic and intrinsic activity, postnatal circuits are largely influenced by exogenous activity and experience. A dynamical neuroelectric field maintained by neural activity is proposed as the fundamental information processing substrate of cognitive function. Time series measurements of the neuroelectric field can be collected by scalp sensors and used to mathematically quantify the essential dynamical features of the neuroelectric field by constructing a digital twin of the dynamical system phase space. The multiscale nonlinear values that result can be organized into tensor data structures, from which latent features can be extracted using tensor factorization. These latent features can be mapped to behavioral constructs to derive digital biomarkers. This computational framework provides a robust method for incorporating neurodynamical measures into neuropsychiatric biomarker discovery.

## Introduction

August Comte, the 19th century French mathematician and philosopher of science, declared that proper research in the physical sciences was based on experimentation. Astronomy, however, was limited only to remote observations and thus, “we can imagine the possibility of determining the shapes of stars, their distances, their sizes, and movements. But there is no means by which we will ever be able to examine their chemical compositions”^1^. Unknown to Comte, characteristic spectral lines had already been observed in telescopic images of the stars by the German chemist Fraunhofer. By the time quantum theory provided the physical laws to explain the stellar spectral lines, the experimental science of astrophysics was emerging. Electromagnetic radiation from the farthest reaches of the universe could be analyzed using physical laws to create detailed models of astrophysical phenomena and to determine the chemical composition of stellar objects and details about physical processes within stars.

A recent proposed framework for psychiatric nosology proposes a computational model that links “underlying dimensional constructs with categorical constructs and actions”^2^. Although neural circuits are explicitly identified as the bridge linking genetics and fundamental neurobiology to behavior, this neural circuit bridge is thought to be forever hidden and thus not computable in models of mental health or dysfunction^3^. We propose that, like astrophysics, measurements may be used to infer remote properties if appropriate physical models are used. The electromagnetic field produced by neural activity and other physiological sources, such as glia, is a dynamical system and thus can be analyzed and quantified using methods from dynamical systems theory. Data from time series measurements provided by scalp electroencephalogram (EEG) recordings are sufficient to reconstruct the essential functional dynamics of the system that produced those signals. A model of neural information processing based on dynamical systems theory can enable essential aspects of neural function to be inferred and mapped to behavioral constructs, much like spectral analysis is used to infer the properties of stars.

The brain has been called a complex dynamical system^4^ This statement typically refers to the topological complexity of the network of neurons that comprise the brain. We suggest that the neuroelectric field produced by the synchronized activity of neurons and related physiology is the complex dynamical system that is the substrate for all neural systems’ functions^5^. The neuroelectric field is the receptor of all sensory input and the physical effector of all movement and behavior. A simple illustration of this is provided by transcranial magnetic stimulation (TMS). A type of “magnetic wand” is held above the scalp to change the neuroelectric field below the scalp, resulting in movements or sensations appropriate to that region of the brain. Change the neuroelectric field, change the mind itself. Each neuron is now recognized as a complicated computing device, further supporting the concept of the neuroelectric field as the substrate for all cognitive activity^6^. This field concept extends even to “simpler” organisms: the unicellular Euplotes has been shown to regulate its membrane potential, an electric field, to enable real-time control over its motor functions^7^

Many mental, neurological, and neurodevelopmental disorders have been described as dynamical diseases, including, but not limited to, epilepsy^8–10^, schizophrenia and bipolar disorder^11–14^, autism^15–18^, disorders of consciousness^19,20^, and Alzheimer’s Disease^21–23^. The conceptualization of brain disorders as dynamical disorders is consistent with the view of the neuroelectric field as the physical substrate of cognitive activity or the mind. The neuroelectric field is a spatially continuous dynamical system^24,25^ The components that contribute to the neuroelectric field include the neurons^26,27^, glia, and other physiological processes that modulate bioelectric fields^28–30^. This perspective is a theoretical framework that considers the neuroelectric field, coupled with neurophysiology, sensory input, and motor output, to be a dynamical system of embodied cognition from which behavior is generated by activation patterns in the continuously evolving neuroelectric field as it interacts with its environment, as well as by internal neuronal interactions^31^. Neural activation patterns may be described mathematically as a dynamical system as they evolve over time^32–34^.

A dynamical system is formally a mathematical system of continuous or discrete equations that describe the evolution of a coupled set of variables over time. The term may also refer to a physical, chemical, biological, or social system that can be modeled by equations that meet the mathematical definition of a dynamical system^35–37^. A dynamical system is capable of information processing, and the information processing capacity of a dynamical system is related to the complexity of the dynamical system^34–38–39^. This principle is now being exploited to build a new generation of information processing or AI systems in the form of physical reservoir computers^40,41^. The analysis of reservoir computing systems may provide additional theoretical concepts for describing the neuroelectric field using dynamical system measures^24,25^.

## Phase Space, Trajectories, Phase Portrait

The state of a dynamical system at any given time can be represented abstractly by a vector in a high-dimensional phase space. For example, if the potential of every one of the 80 billion or so neurons in the brain was known at this moment, the set or vector of 80 billion potential values represents the current state of the brain. A moment later, the potential values of some neurons will have changed, and the new values represent another state. Although the neuroelectric field is continuous, a fine-grained representation of the state of the entire neuroelectric field at, for example, each neuron’s location, can be written as a vector of N real numbers, where N may be approximately 80 billion. Fortunately, such a fine-grained representation is not likely necessary for capturing neural correlates of behavior or markers of neuropathology.

**Figure 1:**
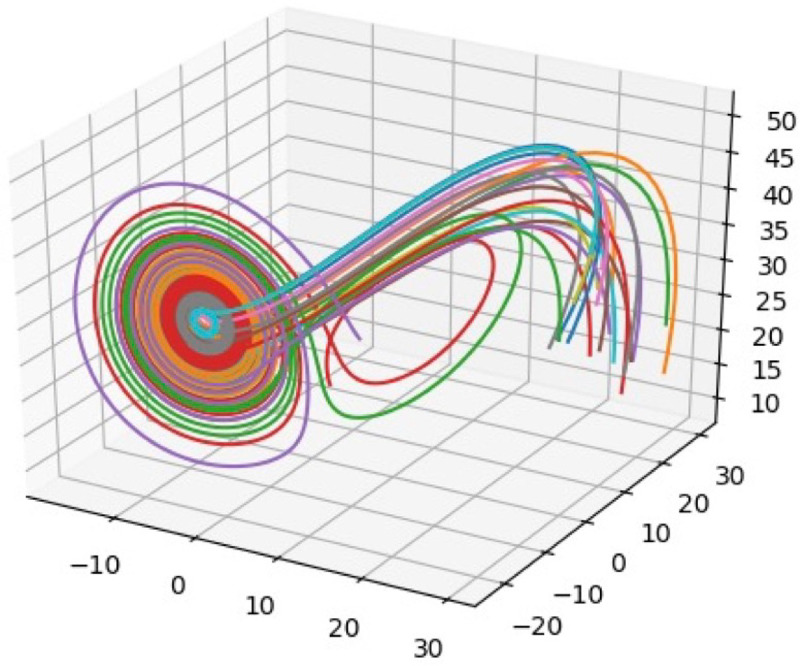
Attractor illustration. The 3-dimensional phase space shows the phase portrait for the Lorentz equations, which were solved numerically in discrete time. The different colored lines each start from a different initial point. The trajectories all converge to the attractor.

The sequence of state vectors through time comprises the trajectory of the neural system. The set of all possible trajectories through neural phase space is called the phase portrait of the system and is an abstract representation of all the possible sequences of the system. The trajectory shown in Fig. 1 is an example of a 3-dimensional phase portrait for the Lorentz system, defined by a specific set of three differential equations. In this example, the phase space is represented by Cartesian coordinates (x,y,z), where x, y, and z are any real numbers, and the phase portrait is embedded in this space. Regardless of the starting point chosen, the trajectory settles into the butterfly-like pattern within the phase space, called an *attractor*. The colored lines show the trajectory of this system from different starting points and collectively create a *phase portrait*.

The phase portrait fully describes how the system may change over time. The set of trajectories shows geometrically, without an explicit time variable, the progression of states through which the system can change. It is like showing a series of time-lapse photographs of the arm of a person throwing a ball. The arm moves smoothly through a certain path and cannot just randomly jump from place to place. The phase portrait is a structural or geometrical representation that completely describes the dynamics of the system. Time is not explicitly present. The phase portrait is a mathematical abstraction that can be analyzed and quantified in more than three dimensions.

Remarkably, time series measurements of any component of a dynamical system, or any linear combination of system components, may be used to reconstruct the essential features of the phase portrait of the entire system – a digital twin – from which quantitative properties of the system may be computed. These properties are called *dynamical invariants*. This concept was first proved mathematically in the *embedding theorems*^42,43^; soon after, computational methods began to be developed for computing dynamical invariants from recurrence plot images that were derived from time series measurements^43–46^. The implications of the embedding theorems and computational methods are profoundly important for EEG measurements but have yet to be fully exploited. EEG sensors provide time series measurements of the neuroelectric field that contain information that enables the phase portrait of the neural system to be reconstructed and quantified. These data contain neural circuit information that can be exploited to build the bridge from neurophysiology to behavior.

**Figure 2:**
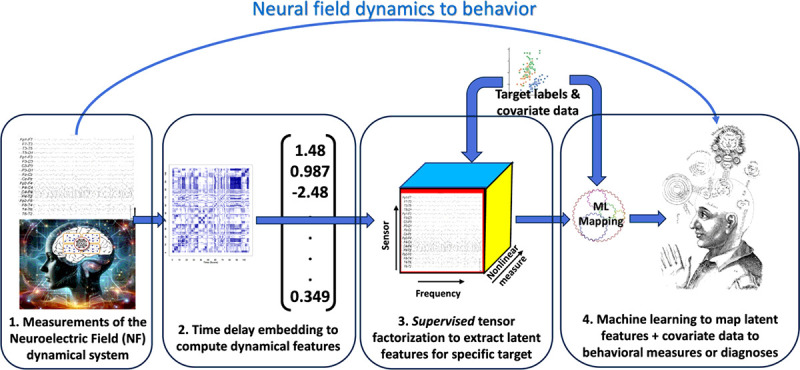
Data processing pipeline from brain function to behavior. (1) EEG signals from multiple sensors measure the neuroelectric field. (2) Dynamical properties are computed for multiple frequency bands from each sensor using methods from dynamical systems theory. (3) Quantitative measures are organized into tensor data structures, from which latent features relevant to the target condition Supervised tensor factorization is used to extract latent features that are most relevant to the outcomes of interest. (4) Latent features and additional data are input to machine learning classifiers or regression algorithms to complete the mapping from EEG time series measurements to behavioral constructs. Outcomes may be binary outcomes or risk probabilities that change over time.

It is well-known that artifacts from eyeblinks or other sources of noise can affect spectral power. A thorough study of the effects of various noise sources on nonlinear dynamical invariants has yet to be done. Figure 2 illustrates the conceptual framework for deriving functional information from EEG measurements of neuroelectric fields, using the measurements to reconstruct the neural phase portrait then mapping the quantitative information extracted from the computed phase portrait to behavior. In short, brain dynamics are measured and mapped to behavior.

We now review some current computational methods to derive dynamical invariants from time series measurements. We further describe how to extract latent dynamical features from multiscale dynamical invariants and map these to behavioral measures, neurological diagnoses, or psychiatric constructs to derive digital biomarkers.

## Results

### Multiscale and Multifrequency Decomposition

**Figure 3:**
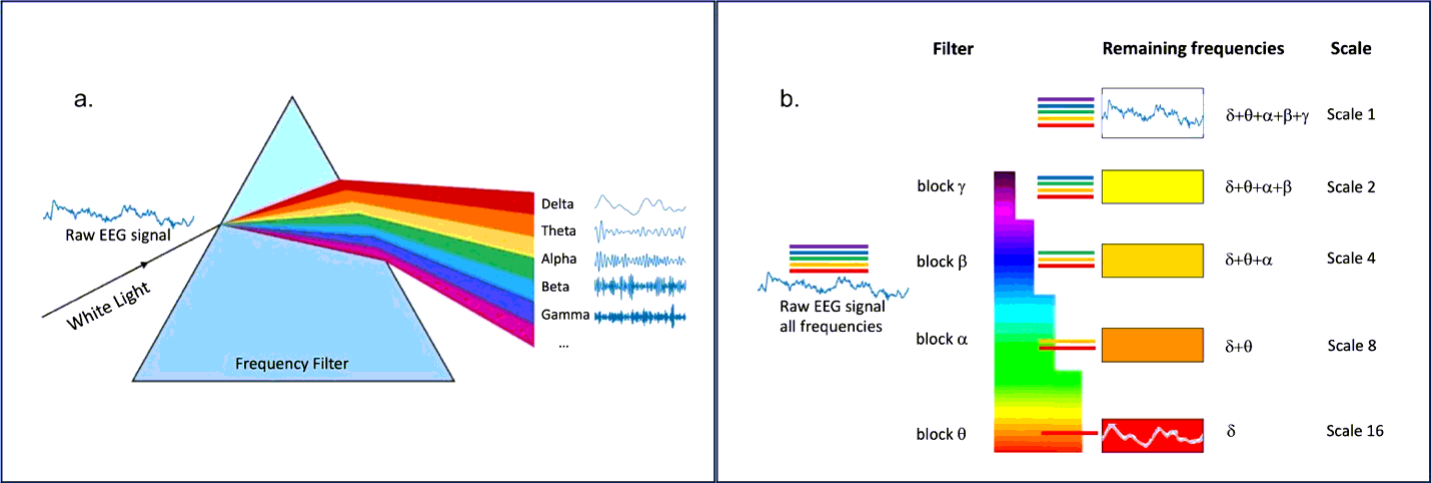
Spectral decomposition (a) and coarse graining (b) are illustrated. Band pass filtering is commonly used to analyze EEG signals by first decomposing the signal into distinct frequency bands using Fourier or related methods. Coarse-graining was introduced in studies of multiscale entropy, where “multiscaling” was accomplished by a coarse-graining algorithm. Coarse-graining is mathematically identical to the “approximations” of the Haar wavelet transform and is low-pass filter, eliminating all frequencies above a specified cutoff.

Physiological neural networks exhibit structure across many scales. Because of this multiscale structure, the electrical fields generated by neural circuits span many scales or frequency bands^61^. Multiscale entropy analysis introduces a scale-dependent approach to nonlinear analysis. Multiscale entropy was first introduced to analyze physiological signals associated with heart disease^47–49^. Although the use of entropy as a measure of physiological complexity was not new, the primary innovation here was recognizing that complexity across multiple scales contained important diagnostic information not available in the raw signal. In general, complexity loss was associated with aging or pathological conditions, with the degree of loss varying across frequency bands or scales^50–52^. The scales produced by the coarse-graining procedure in the multiscale entropy literature have been shown to be identical to the approximations produced by the Haar wavelet transform^53^. This insight has shown the relation between coarse grain scales and the standard frequency bands typically used for EEG spectral power analysis (delta, theta, alpha, beta, gamma). The relation between spectral decomposition and the low-pass filtering implemented by the coarse-graining procedure is illustrated in Fig. 3. In the examples below, we use wavelet details, equivalent to traditional frequency bands, for frequency decomposition or “multiscale” analysis.

### Recurrence Plot Analysis

**Figure 4:**
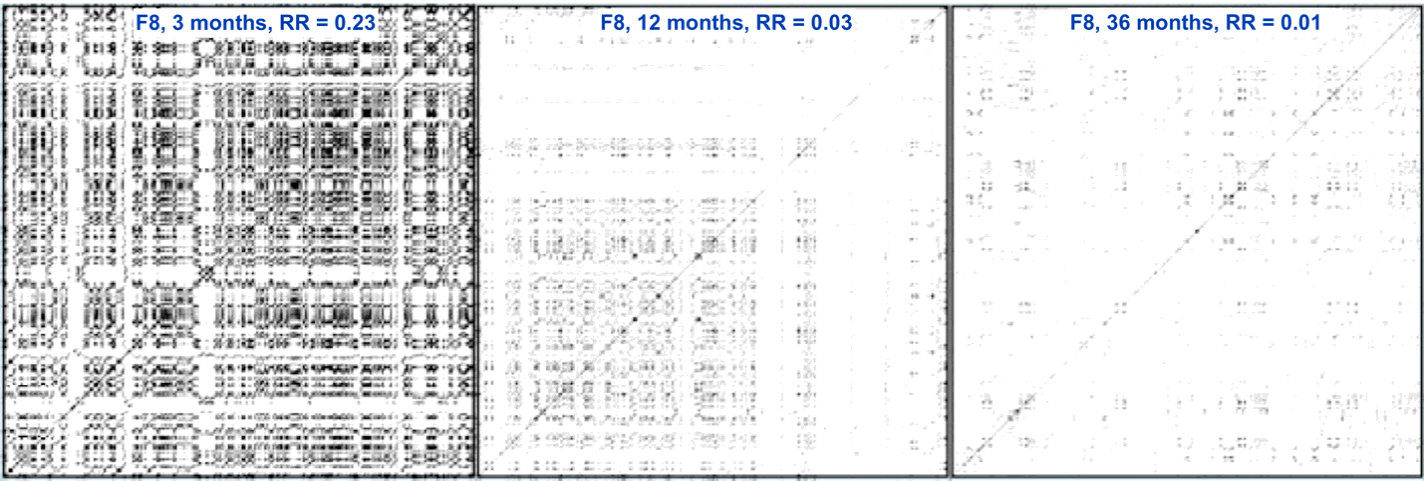
Recurrence plot examples. Recurrence plots derived from an EEG sensor placed on the right lateral prefrontal cortex (F8). Left panel is from an infant at age 3 months; middle panel is from same infant at age 12 months; right panel is from same infant at age 36 months.

Our analysis aims to use EEG signals to infer dynamical properties of the neuroelectric system that produced the signals. Several methods have been developed for computing signal properties. Recurrence plots (RPs) were first introduced as a means to visualize the phase portrait of a dynamical system projected onto a two-dimensional plane^42^. In principle, the RP contains all the essential dynamical system properties from which it was derived^46^. The availability of desktop computers allowed numerical computations that soon resulted in recurrence quantitative analysis (RQA), which was an attempt to quantify the essential properties of a dynamical system from a(n abstract) reconstructed phase portrait. The development and application of RQA to various physical and biological systems suggested that RPs contained essential information to enable quantitative descriptions of dynamical system properties^54^. Examples of recurrence plots derived from time series produced by several different dynamical systems are shown in Fig. 4. The RP might be considered a type of Quick Response (QR) code for brain function.

An advantage of recurrence plot analysis is that it can provide dynamical information even for short, noisy, non-stationary time series^54,55^. For EEG analysis of brain function, Takens’ Theorem (one of the Embedding Theorems) has been shown to be a special case of a more general reconstruction process from multiple time series, which may result in a more accurate reconstruction when time series are digitized or noisy. The results apply to situations having parallel time series measurements for variables related to the same dynamical manifold^56^. This is precisely the situation with multiple scalp sensors on a typical EEG recording.

A consequence of the embedding theorems is that the embedding dimension for the system is determined by the dimension of the system attractors, not by the frequently much higher dimensionality of the microscopic degrees of freedom^57^. Rather, this is a mathematical description of the neurophysiological phenomenon of many neurons synchronizing and firing together to accomplish a given task or behavior.

Dynamical properties of a system can be computed from a recurrence plot by statistically characterizing the various lines and structures in the plot^58–60^. The most common measures and their meaning are summarized in Table 1.

### Recurrence Network Analysis (RN)

A related approach to quantifying the recurrence plot is based on network analysis, where the recurrence plot is interpreted as the adjacency matrix of a complex network^58–61–62^. Recurrence network (RN) analysis exploits an analogy between complex network theory and nonlinear time series analysis^61^. This approach is complementary to RQA, resulting in additional nonlinear information that is not extracted by RQA methods^62^. In particular, the RN approach extracts information regarding the structure of the underlying chaotic attractors, which are not available using the conventional algorithmic methods of nonlinear time-series analysis^63^. Values computed from a recurrence network assess properties of system attractors using network measures, including the ε-clustering coefficient; mesoscopic measures, such as ε-motif density; path-based measures, such as ε-betweennesses; and global measures, such as ε-efficiency^63,64^.

### Power and Other Nonlinear Dynamical Measures

The term “quantitative EEG analysis” has traditionally been defined by the neurology and neurophysiology communities as “the mathematical processing of digitally recorded EEG to highlight specific waveform components, transform the EEG into a format or domain that elucidates relevant information, or associate numerical results with the EEG data for subsequent review or comparison”^65^. Power on multiple frequency bands (“spectral power” or, equivalently, “multiscale power”) has been valuable for inferring diagnostic information. It may be included in the list of signal features.

One of the first applications of nonlinear signal analysis to a physiological system was multiscale entropy to analyze electrocardiograms or heart electrical signals^66^. The specific “entropy” value used was sample entropy, related to the original information entropy proposed by Claude Shannon^67,68^. Since then, the number of different algorithms to compute an entropy value has grown to dozens, including Sample, Approximate, Renyi, and Fuzzy entropies, to name a few^69^. Each has been used for many scientific applications, adding to the confusion over what entropy means^70–72^. Entropy has a physical definition (i.e., the amount of energy in a system that is unavailable to perform work) and a mathematical definition used in information theory^73^, initially defined by Shannon^67^. The mathematical definition has the same form as that used in thermodynamics, and both represent a measure of randomness, though the thermodynamic and mathematical applications are unrelated. In keeping with the goals of this paper, we interpret signal complexity or entropy as a quantitative measure of only *one* dynamical aspect of the system that produced the signal.

In addition to the many entropy variations, several other dynamical properties can be computed from physiological signals. These include correlation dimension^74^, Hurst Exponent, Lyapunov exponents, and Detrended Fluctuation Analysis^75,76^. This is not an exhaustive list, nor should we assume that all quantitative measures of dynamical system properties have been discovered. A remaining challenge is determining the minimal, complete set of measures to fully describe a complex system’s dynamics or phase portrait.

The many dynamical values described thus far are not entirely independent, and more may yet be discovered. As noted previously, many different entropies are defined, including different analytical formulas and methods for estimating entropy from recurrence plots. To allow for possible overlap among measures and to retain structural relations between different sensors or frequency information for adjacent bands, a multiarray or tensor representation is proposed for further analysis and to extract a smaller set of latent dynamical features. This approach allows a low dimensional representation of the multifrequency, multisensor data to be extracted using supervised tensor factorization methods. The resultant latent features in the low dimensional representation can be used as biomarkers in machine learning or regression models.

### Tensor Representation of Multiscale EEG Features

**Figure 5:**
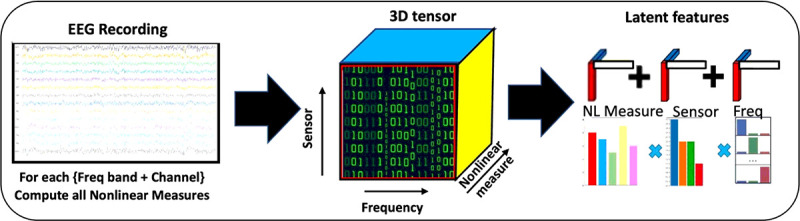
Tensor representation of multifrequency, multisensory, dynamical measures.

The dynamical analysis presented results in hundreds or thousands of values derived from each EEG recording. Typical values are 19 or more sensors, 6 frequency bands, and 15 dynamical measures (shown in Table 1), which yield 1710 dynamical values. High-density EEG nets used in research may have as many as 256 sensors, which would result in tens of thousands of dynamical values. These values are likely not entirely independent. For example, nearby sensors are likely to have some sources in common. Adjacent frequency bands are likely to have more in common than those farther apart. Moreover, the minimum set of dynamical measures necessary to characterize a system is not yet known. The set of EEG measures described above can be arranged into a tensor, i.e., a 3-dimensional data structure (sensor, frequency band, nonlinear measure), as illustrated in Fig. 5. The data from multiple patients or research participants can be represented as a fourth axis, creating a fourth-order tensor of dimensions N × Ns × Nf × Nm, where N is the number of participants. The cubes in Fig. 5 illustrate the structure of EEG features. Tensors have been used to represent EEG features recently^77^, but *supervised* tensor factorization has not been proposed in this context previously.

The goal of tensor factorization is to identify a small number of latent variables within the raw data that explain most of the variation in the data. Importantly, the predominant variations in the data may not be the most highly correlated with target conditions of interest. Thus, supervising on a primary target can improve the value of the identified factors. Further, often in clinical contexts, the latent variables are influenced by additional variables, such as environmental exposures or gender assigned at birth. Supervising on a primary target variable and additional covariate variables can improve the accuracy and usefulness of the discovered latent variables^78^.

A supervised tensor decomposition algorithm, such as the Canonical Polyadic (CP) regression algorithm in the Tensorly Python package^79^ or the SupCP described in the literature^80^, can extract latent features that are most aligned with the target and covariate variables. Tensor factorization is analogous to Singular Value Decomposition (SVC) for matrices but generalized to multiple dimensions and, again in the algorithms presented here, *supervised*, allowing the target outcomes to influence the factor extraction process. The extracted latent factors are analogous to principal components found by Principal Components Analysis (PCA) used for matrix analysis. The result of supervised tensor factorization is the reduction of 1710 (or more) dynamical values to a much smaller set of perhaps 3 to 20 factors relevant to the condition of interest. These factors may be used as the fundamental features to train a machine learning model or statistical forecast model.

An important feature of tensor factorization is that the latent factors are interpretable. Supervised tensor decomposition can extract latent features and explore the underlying neurophysiology 79–81 relevant to the labels or categories used for the supervised factorization. Like the Singular Value Decomposition (SVC) for matrices, a tensor decomposition will find a reduced dimensionality feature set^81–83^. The canonical polyadic (CP) decomposition of a rank R tensor factorizes the tensor into a sum of *R* rank-1 tensors^84^. Our approach to tensor factorization implements a supervised version of the CP factorization in which covariates inform the latent variables. The covariates include the outcomes of interest, extracting latent structures - biomarkers - that are more accurate and interpretable than unsupervised factorization provides. Moreover, these latent factors represent dynamical information that is derived from the dynamical neuroelectric field, thus representing the underlying brain physiology.

## Example

### Neurodevelopment: Calendar Age Correlation with Latent Dynamical Features

The process of neurodevelopment is one of growth followed by pruning of neural structures, enabling an increasingly complex neuroelectric field to be maintained. Our example is intended to demonstrate that latent dynamical factors correlated with calendar age can be found using the processing procedure illustrated in Fig. 2.

This analysis used data from the Emotion Project (EP), an ongoing longitudinal cohort study on the development of emotion processing and mental health from infancy through childhood in a community sample. Detailed information about participants in the EP is published elsewhere^80^. Participants were recruited at 5-, 7-, or 12-months of age (infancy assessment), with additional data collected when the children were 3, 5, and 7 years of age. Segments of awake, resting state EEG recordings collected at each of these time points were used for our analysis. This example is chosen to demonstrate how dynamical values as described above, together with supervised tensor factorization, can be used to find latent variables relevant to a specific target. The target in this example is calendar age, which is associated with increasing cognitive ability in typically developing children.

**Figure 6:**
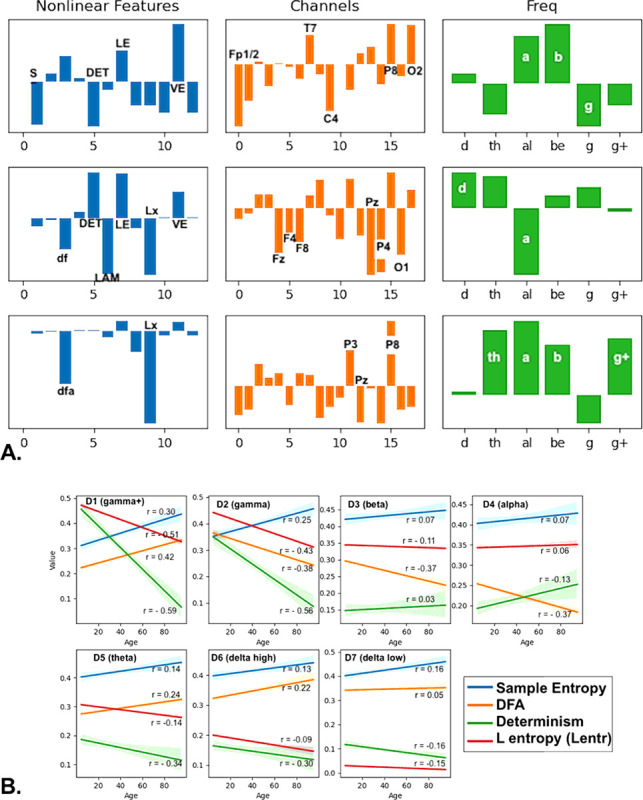
Neurodevelopment and dynamical invariants or nonlinear measures. 6.A shows the latent factors that were extracted in the supervised tensor factorization process. The bar graphs reveal the relative contributions of the nonlinear measures, channel locations, and frequency bands. With this information, the specific measures can be further explored and analyzed, as shown in 6.B, where four of the measures are averaged over all sensors and plotted against age.

EEG time series from 350 subjects were analyzed, with 19 sensors chosen from the standard 10–20 montage, decomposed into 6 frequency bands for each recording. Power and the dynamical invariants described in Table 1 were computed and organized into a tensor structure. Supervised tensor factorization provided by the regression function in the Python tensorly package (https://tensorly.org/) was used to extract three latent factors in a 5-fold cross-validation scheme, with calendar age as the target independent variable. Independent latent factors were computed for each fold. We found that the latent factors were highly correlated with age in the test sets (R = 0.77, p<1.0e-60) when supervised factorization was used, but only weakly correlated (R = 0.24, p = 2.0e-7) when unsupervised factorization was used to extract latent factors. Results for calendar age prediction from EEG are shown in Table 2. The three latent factors extracted are illustrated in Fig. 6.A, and plots of four prominent nonlinear measures in each frequency band are shown in Fig. 6.B, averaged over all sensors.

We found that three latent EEG factors together are strongly correlated with calendar age (r = 0.79, Table 2). The relative contributions of the raw EEG measures are shown in the bar graphs of the latent factors that were extracted in the supervised tensor factorization process (Fig. 6.A). Four measures are averaged over all sensors and plotted against age (Fig. 6.B) to view changes over developmental time. These plots illustrate how the nonlinear measures can be used and evaluated when comparing different groups or variations in continuous variables of interest, such as developmental age. Importantly, we found that when unsupervised tensor factorization was used to extract latent variables, the correlation with calendar age was considerably less.

## Discussion

Our primary goal was to present a dynamical systems model of brain function based on a neuroelectric field as the fundamental substrate of cognition and behavior. This model suggests a computational framework for quantitatively characterizing brain function using time series (EEG) measurements of the neuroelectric field to compute dynamical properties or ‘invariants’. Supervised tensor factorization is presented as a general methodology for organizing these EEG-derived values, allowing for inclusion of new nonlinear measures in future research. Our goal here is clinically pragmatic: The neuroelectric field can be measured by EEG sensors, characterized by the tools of dynamical systems analysis, and mapped to cognitive constructs, developmental behavioral milestones, or psychiatric conditions using supervised tensor factorization and machine learning. That is, the presented framework is intended as a general computational approach for neuropsychiatric biomarker discovery and evaluation.

Nonlinear analysis of EEG time series has been used for several decades to compute signal properties to be used as biomarkers for brain disorders. However, a comprehensive perspective that seeks to find a minimal set of measures, a *basis set* in mathematical terms, that comprehensively characterizes (brain) system dynamics has yet to be presented. As we described above, the number of nonlinear measures that can be computed continues to increase, with more than 20 different entropies alone being defined^69^. We present a flexible framework that allows new nonlinear measures to be added, with the same supervised tensor factorization scheme to extract a smaller, latent set of factors most correlated with a chosen target, such as a specific disease or psychiatric construct.

Artifacts and noise in EEG measurements are known to affect spectral power values. For example, eyeblinks and facial muscle movements cause large deviations in signals from sensors near the activity from these non-neural sources. Noise introduced to the measurement and digitization process may significantly affect power values. An important question yet to be answered is how much these artifacts affect nonlinear measures. It may be that some dynamical measures are relatively immune to transient, large amplitude muscle artifacts. On the other hand, small amplitude noise, which may have little effect on power, may significantly alter values of entropy or other dynamical measures. Importantly, the effects of filtering for noise, artifacts, or frequencies on the various dynamical measures is unknown. Since filtering is commonly applied to EEG signals, a systematic study of these effects is important for further development of dynamical analysis to neural biomarker applications.

We hope that researchers will contribute to this endeavor to find a basic set of nonlinear measures for neuroscientific research and clinical neurophysiology practice. Our multiscale nonlinear signal analysis framework and supervised tensor methods for extracting latent factors will enable expert researchers in various fields to explore the nonlinear dynamics of the brain relevant to their specialties. In so doing, the community will increasingly refine a comprehensive set of measures and computational methods.

The calendar age predictions shown in Table 2 illustrate that supervised learning is required for at least some prediction targets. The poor result with unsupervised factors suggests that the variation in EEG dynamical measures is large in this population independent of age; thus, supervision helps to identify latent factors relevant to age. A correlation coefficient for an objective measure of functional brain development might be expected to be higher than the correlation with age alone. This is an open topic for further research. An EEG-based biomarker for measuring brain function during development might be useful clinically for identifying those who are falling behind typically developing peers. The inclusion of sex as a covariate did not affect the correlation coefficient with calendar age in this study cohort examined from infancy to age 7 years. Our framework enables other covariates, such as genetics or environmental exposures, to be studied for their effect on outcomes.

Future mathematical and computational research is needed to extend the framework presented. Identification of a minimal comprehensive set of measures that should be computed from every EEG recording would establish a common set of measures for use by the entire neurophysiology community to create a shared learning neuropsychiatric data system.

The computational tools presented have been useful for discovering potential digital biomarkers for many neuropsychiatric disorders, some of which we have explored in our own research. Application, analysis, and clinical testing are needed for specific disease cases to build machine learning models derived from training data. This will require specialists from many fields, using their knowledge to properly train as well as upgrade the model framework as needed.

Research to advance supervised tensor factorization to include additional data and developmental trajectories will enable multimodal data fusion^85,86^ allowing personalized analysis and clinical decision support. We believe that the theoretical and computational framework presented here will enable new exploration to discover clinically useful biomarkers for monitoring many neuropsychiatric disorders.

## Figures and Tables

**Table 1:** A summary of the most common dynamical features computed from time series. Each measure may be computed for the time series from each EEG sensor and may also be computed for sub-signals that represent defined frequency bands, such as the traditional delta, theta, alpha, beta, and gamma bands used in clinical neurophysiology.

Nonlinear Invariant Variable	Description
Power	**Power** is a measure of how large the waves in a signal are. Specifically, it is the mean of the squared amplitude over any interval. Spectral power is a common measure of the mean power within defined frequency bands.
**Recurrence Quantitative Analysis values computed from Recurrence Plots**	
Python software: pyunicorn package, http://www.pik-potsdam.de/~donges/pyunicorn/	
Recurrence Rate (RR)	**Recurrence rate** is the density of recurrence points in a recurrence plot and corresponds with the probability that a specific state will recur. An option used for our calculations is to set a constant RR (typically 0.05, which was used for calculations presented in this paper) rather than a threshold for coloring points to create the recurrence plot.
Determinism (DET)	**Determinism** is related to dynamical system predictability and is derived from diagonal lines in the recurrence plot as an indicator of deterministic behavior, with a value between 0 and 1. A purely sinusoidal signal will have a value of 1, and a purely stochastic signal will result in a value close to 0.
Laminarity (LAM)	The concept of **laminarity** is a generalization of laminar flow in fluid dynamics. It is related to the amount of laminar or smooth phases in the system and intermittency or alternation between periodic and chaotic regimes.
Line Length Entropy (Lentr)	**Line Length Entropy** is the Shannon Entropy of the diagonal line lengths in the recurrence plot and reflects the complexity of the deterministic structure in the system. Although not identical, it represents a similar measure to Sample Entropy, described below.
Mean Line Length (Lmean)	**Mean Line Length** is the time that two segments of the recurrence plot trajectory are close to each other and can be interpreted as the mean prediction time of the signal, a measure of chaos or divergence from an initial point.
Maximal line length (Lmax)	**Maximal Line Length** is the length of the diagonal lines related to how long segments of the phase space trajectory run parallel, i.e. on the divergence behavior of the trajectories. This concept is related to the maximum Lyapunov exponent, but the two are not exactly equivalent. Lmax describes the divergence of trajectories with small differences in initial states. The higher Lmax, the greater sensitivity to initial conditions, and the less predictable signal behavior.
Trapping time (TT)	**Trapping time** is an estimate of the time that a system will remain in a given state, such as the length of transition states, as opposed to the time for the transition to take place.
Vertical Entropy (VertEnt)	**Vertical Entropy** is defined as the entropy of the probability to find a vertical line of exactly length l in the recurrence plot. It reflects the complexity of the recurrence plot with respect to vertical lines.
Average Vertical Entropy of White lines (AvgWhiteVertEnt)	**Average Vertical Entropy of White Lines** is another sample entropy value computed from the average white vertical line length distribution, also called Mean Recurrence Time. The relationship or correlation in specific datasets between AvgWhiteEnt, Lentr, and the entropy values discussed below have not yet been studied.
**Complexity measures computed directly from a time series**	
Python software: nolds package (https://pypi.org/project/nolds/)	
Entropies	**Sample entropy**, perhaps the most used entropy measure in physiology, measures the “complexity” of a time series, though the meaning of this is elusive. It is defined as the negative natural logarithm of the conditional probability that two sequences similar for *m* points remain similar at the next point, where self-matches are not included in calculating the probability. Its computation is based on approximate entropy, but reduces bias and relative consistency, while being largely independent of signal length. A lower value of sample entropy indicates more self-similarity in a signal and lower complexity. Algorithms for computing these may be found in the publicly available nolds package. Python packages for three other entropy measures that have been found useful as epilepsy biomarker candidates, fuzzy entropy^28^, permutation entropy^29^, and approximate entropy^30^ (EntroPy), are readily available and will also be included.
Correlation Dimension (CD)	**Correlation dimension** is a measure of the fractal dimension of the phase space of a dynamical system, derived using the Grassberger-Procaccia algorithm. It gives an estimate for the number of active degrees of freedom, which is difficult to ascertain from other quantities (Kantz & Schreiber, 1995). Physiological data generally are aperiodic, which can be caused by chaotic deterministic dynamics. Low dimensional strange attractors are one possible signature of deterministic chaos. Such systems are often characterized by only a very few degrees of freedom, even if the true phase space may possess a very high dimension.
Detrended Fluctuation Analysis (DFA)	**Detrended Fluctuation Analysis** is a method for determining the statistical self-affinity of a signal. That is, DFA is useful for analyzing time series that appear to be long-memory processes or 1/f noise. The obtained exponent is like the Hurst exponent, except that DFA may also be applied to non-stationary signals. This method has been used on control time series that consist of long-range correlations with the superposition of a non-stationary external trend and has been successfully applied to detect long range correlations in highly heterogeneous DNA sequences and other physiological applications (Peng et al., 1995).
Hurst Exponent	The **Hurst exponent** is a statistical measure of long-term memory of time series, like dFa. The Hurst exponent is a statistical measure used to characterize the self-similarity and long-term memory properties of time sequences. His directly related to fractal dimension, *D,* and is a measure of a data series’ “mild” or “wild” randomness (Mandelbrot & Hudson, 2006). As a general rule for interpretation, value of H = 0.5 corresponds to a random walk, H < 0.5 indicates a time series with long-term switching between high and low values, and H > 0.5 indicates a time series with long-term positive autocorrelation (Zhang et al., 2023). In the context of physiological signals, it can help in understanding the patterns and predictability of biological processes over time (Zhang et al., 2023). In the analysis of EEG signals, the Hurst exponent has been used to characterize the signal as either mean-reverting, trending, or a random walk (Natarajan et al., 2004).
Lyapunov exponent	The **Lyapunov exponent** provides insights into the stability of dynamical systems by quantifying how quickly nearby trajectories in the phase space diverge or converge over time. A positive Lyapunov exponent indicates chaotic behavior (divergence), where small initial differences grow arbitrarily large. Conversely, a negative exponent suggests convergence to periodic trajectories (an attractor). The Lyapunov exponent is typically computed from the Jacobian matrix, which describes how small perturbations evolve in the system (Hilborn, 2017).

**Table 2:** Regression to predict calendar age from EEG complexity for three datasets: Emotion Project (EP), Infant Siblings Project (ISP), and combined EP + ISP. Two different regressor algorithms were used with five-fold cross validation in every case. Tensor rank = 3 (3 latent factors); Random Forest and Gradient Boost regressors from (Python scikit-learn package: https://scikit-learn.org/stable/modules/generated/sklearn.ensemble.RandomForestClassifier.html)

Data set	Regressor	Supervised Factorization, with sex as covariate		Unsupervised, with sex as covariate to ML step		Unsupervised Factorization	
		Corr coeff (r)	Corr coeff (r)	Corr coeff (r)	Corr coeff (r)	Corr coeff (r)	Mean abs error (mths)
**EP** N = 350	Random Forest (RF)	0.76	12.2	0.34	20.9	0.13	24.2
5 to 84 months	Gradient boost (GB)	0.77	12.1	0.26	21.6	0.29	21.2

## Data Availability

The data used in this study were consented specifically for use by researchers affiliated with Boston Children’s Hospital. For this reason, the EEG and patient data cannot be released publicly at this time. However, the computational framework is designed to be used with any available EEG datasets, many of which are available on public sites.
